# Validierung eines Modells zur Vorhersage des Sprachverstehens nach Cochleaimplantation

**DOI:** 10.1007/s00106-023-01284-z

**Published:** 2023-03-21

**Authors:** Ulrich Hoppe, Anne Hast, Thomas Hocke

**Affiliations:** 1 Audiologische Abteilung, Hals-Nasen-Ohrenklinik, Kopf- und Halschirurgie, Universitätsklinikum Erlangen, Waldstr. 1, 91054 Erlangen, Deutschland; 2 Cochlear Deutschland GmbH & Co. KG, Mailänder Str. 4a, 30539 Hannover, Deutschland

**Keywords:** Sprachdiskriminationstest, Sprachaudiometrie, Hörtests, Hörverlust, Hörgeräte, Speech discrimination tests, Speech audiometry, Hearing tests, Hearing loss, Hearing aids

## Abstract

**Hintergrund:**

Wird mit konventionellen Hörsystemen kein ausreichendes Sprachverstehen mehr erreicht, ist eine audiologische Indikation für eine Versorgung mit einem Cochleaimplantat (CI) gegeben. Für die CI-Versorgung gibt es bisher jedoch keine etablierten Zielkriterien für das zu erreichende Sprachverstehen. Ziel dieser Studie ist es, ein bereits bestehendes Vorhersagemodell für das Sprachverstehen nach CI-Versorgung zu validieren. Dieses wird auf verschiedene Patientengruppen angewendet.

**Material und Methoden:**

In die prospektive Studie wurden 124 postlingual ertaubte erwachsene Patienten eingeschlossen. Das auf präoperativem maximalem Einsilberverstehen, dem Einsilberverstehen mit Hörgerät bei 65 dB_SPL_ und Lebensalter zum Zeitpunkt der Versorgung basierende Modell wurde hinsichtlich der Vorhersagegenauigkeit für das Einsilberverstehen mit CI nach 6 Monaten untersucht.

**Ergebnisse:**

Das Sprachverstehen verbesserte sich im Mittel nach 6 Monaten von 10 % mit Hörgerät auf 65 % mit CI, einhergehend mit einer statistisch signifikanten Verbesserung für 93 % der Fälle. Eine Verschlechterung des versorgten unilateralen Sprachverstehens wurde nicht beobachtet. Der mittlere Vorhersagefehler lag in den Fällen mit präoperativem residualem Einsilberverstehen bei 11,5 Prozentpunkten und in allen anderen Fällen bei 23,2 Prozentpunkten.

**Schlussfolgerung:**

Auch bei Patienten mit mittel- bis hochgradiger Schwerhörigkeit und unzureichendem Sprachverstehen mit Hörgerät ist die CI-Versorgung eine Therapieoption. Das auf präoperativ erhobenen Daten basierende Modell zur Vorhersage des Sprachverstehens mit CI kann bei der präoperativen Beratung und im Rahmen der postoperativen Qualitätssicherung genutzt werden.

## Abstract (en)

**Background:**

If sufficient speech discrimination is no longer achieved with conventional hearing systems, an audiological indication for a cochlear implant (CI) is given. However, there are no established target criteria for CI aftercare with regard to the level of speech comprehension to be achieved. The aim of this study is to validate an existing predictive model for speech comprehension after CI provision. This is applied to different patient groups.

**Materials and methods:**

The prospective study included 124 postlingually deaf adults. The model is based on preoperative maximum monosyllabic recognition score, aided monosyllabic recognition score at 65 dB_SPL_, and age the time of implantation. The model was investigated with regard to prediction accuracy for monosyllabic recognition with CI after 6 months.

**Results:**

Mean speech discrimination improved from 10% with hearing aid to 65% with CI after 6 months, with a statistically significant improvement in 93% of cases. Deterioration of aided unilateral speech discrimination was not observed. The mean prediction error was 11.5 percentage points in the cases with preoperative scores better than zero and 23.2 percentage points in all other cases.

**Conclusion:**

Cochlear implantation should also be considered in patients with moderately severe to severe hearing loss and insufficient speech discrimination with hearing aids. The model based on preoperatively measured data for predicting speech discrimination with CI can be used in preoperative consultation and in the context of postoperative quality assurance.

## CI-Indikation

Hauptziel einer apparativen Versorgung schwerhöriger Patienten ist die Wiederherstellung bzw. Verbesserung des Sprachverstehens. Die Versorgung mit schallverstärkenden Hörgeräten ist zunächst die Therapiemethode der Wahl. Erst wenn mit diesen oder anderen Hörsystemen kein ausreichendes Sprachverstehen mehr erreicht werden kann, bestehen die audiologischen Voraussetzungen für die Indikation zu einer Versorgung mit einem Cochleaimplantat (CI). Nach der deutschen S2k-Leitlinie „Cochlea-Implantat Versorgung“ kann diese bei einem Einsilberverstehen mit optimierter Hörgeräteversorgung von bis zu 60 % bei einem Sprachpegel von 65 dB_SPL_ in Betracht gezogen werden [1]. Somit kann auch bei Menschen mit vergleichsweise gutem Tonhörvermögen die CI-Indikation gegeben sein, wenn mit Hörgerät kein ausreichendes Sprachverstehen erreicht wird.

## Quantifizierung des Sprachverstehens

Für die Quantifizierung des Sprachverstehens stehen verschiedene Tests zur Verfügung. Der Freiburger Einsilbertest hat sich als Standardverfahren sowohl in der klinischen Hörgeräte- und CI-Diagnostik als auch in wissenschaftlichen Untersuchungen etabliert [1, 9, 10, 18, 22, 23]. Bei der Hörgeräteversorgung gibt das maximal erreichbare Einsilberverstehen (mEV) Hinweise auf das mit Hörgeräten anzustrebende Sprachverstehen für Umgangssprache und kann als Zielkriterium für die Hörgeräteanpassung verwendet werden [12, 17, 25]. Für die CI-Versorgung und -Anpassung gibt es bisher jedoch keine damit vergleichbaren etablierten Zielkriterien. Ein Grund dafür liegt in der Heterogenität der Patientengruppen, der unzureichenden Bestimmbarkeit der funktionellen Integrität der zentralnervösen Verarbeitung, der unzureichenden Kenntnis entsprechender Einflussfaktoren sowie der Schwierigkeit, selbige in großen klinischen Studien zu kontrollieren [3, 4, 8, 16, 20]. Gerade für Menschen mit noch vorhandenem Sprachverstehen ist die präoperative Abschätzung des mit CI-System erwartbaren Sprachverstehens von besonderer Bedeutung.

In einer früheren Studie [13] wurde gezeigt, dass das präoperativ gemessene mEV als unterer Schätzer für das mit CI erreichbare Sprachverstehen verwendet werden kann. Jüngere Arbeiten bestätigen dieses Ergebnis [14, 27]. Unlängst wurde für Patienten mit Hörverlusten < 80 dB_HL_ ein Vorhersagemodell für das mit CI zu erwartende Einsilberverstehen entwickelt, das auf den präoperativ bekannten Größen mEV, Einsilberverstehen mit Hörgerät bei 65 dB_SPL_, EV_65_(HG), und dem Lebensalter zum Zeitpunkt der Versorgung besteht [14], siehe Gl. 1.

1 $$EV_{65}\left(CI\right)\left[{\%}\right]=\frac{100}{1+e^{-\left(\beta _{0}+\beta _{1}\cdot mEV+\beta _{2}\cdot \textit{Alter}+\beta _{3}\cdot EV_{65}\left(HG\right)\right)}}$$

mit

β_0_ =: 0,84 ± 0,18
β_1_ =: 0,012 ± 0,0015 1/%
β_2_ =: −0,0094 ± 0,0025 1/Jahre
β_3_ =: 0,0059 ± 0,0026 1/%

Aus den Vorzeichen der Parameter ist ersichtlich, dass sich ein höheres Lebensalter negativ auf das Sprachverstehen auswirkt, wohingegen ein höheres mEV oder EV_65_(HG) zu einem höheren Sprachverstehen mit CI führt.

Der mit Gl. 1 bestimmte Vorhersagewert wird derzeit am Universitätsklinikum Erlangen als Parameter für die Qualitätssicherung und für die individuelle präoperative Beratung der CI-Kandidaten verwendet. Insbesondere bei Fällen mit noch substanziellem Restgehör ist eine individuelle Prognose wünschenswert [19, 24, 28]. Ziel dieser Studie ist es, das in einer früheren, retrospektiven Studie entwickelte Modell [14] im Rahmen einer prospektiven Studie zu validieren. Hierzu wurde die Anwendung auf 2 Patientengruppen mit unterschiedlichem präoperativem mEV (mEV = 0 % bzw. mEV > 0 %), unabhängig vom tonaudiometrischen Hörverlust, hinsichtlich des Vorhersagefehlers untersucht.

## Patienten und Methode

Die vorgestellten Daten wurden im Rahmen klinischer Routineuntersuchungen zur CI-Vordiagnostik und der Basis- und Folgetherapie der CI-Nachbehandlung erhoben. Die prospektive Studie wurde von der zuständigen Ethikkommission befürwortet (AZ 60_20B) und beim deutschen Register für klinische Studien angemeldet (DRKS00023351).

### Patientenmerkmale

Insgesamt wurden in dieser Studie die Daten von allen erwachsenen Patienten ausgewertet, welche im Zeitraum Oktober 2020 bis Dezember 2021 in der Hals-Nasen-Ohrenklinik, Kopf- und Halschirurgie des Universitätsklinikums Erlangen mit einem Nucleus-CI (Fa. Cochlear Ltd., Sydney, Australien) versorgt wurden. Einschlusskriterien waren eine postlingual entwickelte Hörstörung, Deutsch als Muttersprache, CI-Indikation entsprechend den aktuellen deutschen CI-Leitlinien [1] aufgrund einer Schallempfindungs- oder kombinierten Schwerhörigkeit und mindestens 6 Monate Rehabilitation im CI-Zentrum der Autoren. Ausschlusskriterium war eine kognitive Beeinträchtigung, die die Durchführung der Sprachaudiometrie beeinflusst hätte. Ebenfalls wurden Patienten mit bereits bestehender ipsilateraler CI-Versorgung (Reimplantation) ausgeschlossen. Derzeit liegen für 124 Patienten sowohl präoperative Daten als auch postoperative Sprachverstehenswerte für einen Zeitraum von mindestens 6 Monaten nach CI-Versorgung vor. Das Patientenkollektiv bestand aus 73 Männern und 51 Frauen. Das Lebensalter zum Zeitpunkt der CI-Versorgung lag im Mittel bei 65,0 ± 13,9 Jahre. Alle Patienten nutzten zum Zeitpunkt der CI-Vordiagnostik ein Hörgerät auf der später versorgten Seite. Der Hörverlust für Luftleitung wurde als Mittelwert über die 4 Oktavfrequenzen 0,5; 1; 2 und 4 kHz bestimmt (4FPTA, „4-frequency pure tone audiometry“). Für Hörschwellen jenseits der maximal möglichen Darbietungspegel der Audiometer wurde ein Wert von 130 dB_HL_ imputiert. Damit ergab sich ein mittlerer Hörverlust von 92 ± 21 dB_HL_. In der Mehrzahl der Fälle handelte es sich um eine unilaterale CI-Versorgung mit einem mittleren tonaudiometrischen Hörverlust auf der kontralateralen Seite von im Mittel 54 ± 26 dB_HL_. Bei 21 Fällen war die kontralaterale Seite bereits mit einem CI versorgt. Als Sprachprozessor wurde von 100 CI-Trägern der Hinter-dem-Ohr-Prozessor (Modell-Nr. CP1000, Fa. Cochlear Ltd., Sydney, Australien) verwendet, 24 Patienten trugen den ohrfernen Prozessor. Die demografischen Details sind in Tab. 1 zusammengefasst.

**Table 1:** 

Demografie								
**Geschlecht**	*Männer*			*Frauen*				
Anzahl (*n*)	73			51				
*–*	*Minimum*			*Maximum*		*Median*		
**Alter (Jahre)**	25			86		66		
**Dauer der Schwerhörigkeit (Jahre)**	0			79		20		
**Dauer der Hörgeräteverwendung (Jahre)**	0			60		10		
**Hörverlust ipsilateral (dB**_**HL**_**)**	47			130		87		
**Hörverlust kontralateral (dB**_**HL**_**)**	3			95		63		
**mEV ipsilateral (%)**	0			100		23		
**EV**_**65**_**(HG) (%)**	0			50		0		
**Ätiologie:**	*Unbekannt*	*Infektion*	*M. Menière*	*Trauma*	*Cholesteatom*	*Ototoxische Medikamente*	*Meningitis*	*Syndromal*
Anzahl (*n*)	84	13	8	8	3	3	2	3
**Implantattyp**	*CI612*			*CI632*		*CI622*		
	32			91		1		
**Prozessortyp**	*CP1000*			*Kanso 1*		*Kanso 2*		
	100			21		3		
**Anzahl der Therapieeinheiten**	*Minimum*			*Maximum*		*Median*		
	6			12		9,5		

*EV*_*65*_*(HG) *Einsilberverstehen mit Hörgerät bei 65 dB_SPL_, *mEV* maximal erreichbares Einsilberverstehen

### Messungen

Es wurden tonaudiometrische Messungen (Luftleitung) und sprachaudiometrische Messungen (Freiburger Einsilbertest, DIN 45621) analysiert. Von den präoperativen Messungen wurden der 4FPTA, das maximale Einsilberverstehen im Sprachaudiogram nach DIN 45621, also das mEV in %, und das monaurale mit Hörgerät im Freifeld gemessene Einsilberverstehen bei 65 dB_SPL_, also das EV_65_(HG) in %, verwendet. Die Hörgeräte wurden vorab technisch überprüft. Insbesondere wurde durch In-situ-Messungen sichergestellt, dass die Einstellungen zu den adäquaten Zielverstärkungen führten [5]. Von den postoperativen Messungen wurde das Einsilberverstehen mit CI-System im Freifeld bei 65 dB_SPL_, also das EV_65_(CI) in %, ausgewertet.

Die Messungen im Freifeld erfolgten in einer schallisolierten Kabine (6 × 6 m). Der Lautsprecher wurde 1,5 m vor dem Patienten (0°-Azimut) platziert. Das kontralaterale Ohr wurde, sofern nötig, regelrecht mit Breitbandrauschen über Kopfhörer maskiert.

### Datenanalyse

Die Analyse und die Erstellung der Abbildungen erfolgte mittels des Softwarepakets MATLAB® R2019b (Fa. MathWorks, Natick, MA, USA). Für die Vorhersage des Sprachverstehens mit CI-System wurden 3 präoperative Größen, also mEV, EV_65_(HG) und Lebensalter (Gl. 1), herangezogen. Der Vorhersagefehler wurde anhand des medianen absoluten Fehlers („median absolute error“, MAE) quantifiziert.

## Ergebnisse

### Präoperative Audiometrie

**Figure 1:**
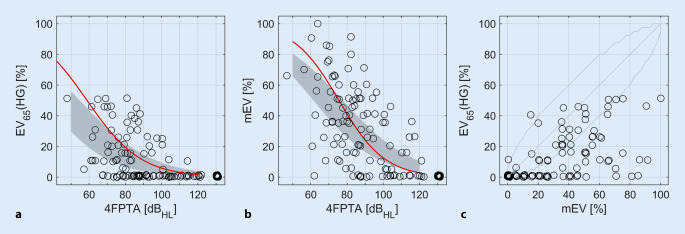

In Abb. 1 werden die Beziehungen zwischen dem 4FPTA, dem maximalen Einsilberverstehen und dem im Freifeld bei 65 dB_SPL_ gemessenen Sprachverstehen mit Hörgerät beschrieben. Die roten Linien (Abb. 1a, b) stehen für das mittlere EV_65_(HG) bzw. das mEV in Abhängigkeit vom 4FPTA aus einer früheren Studie [11] in einer Population von Hörgeräteträgern. Bei allen Fällen lag das EV_65_(HG) kleiner oder gleich 50 % und somit deutlich im Indikationsbereich für die CI-Versorgung [1]. Das mEV lag in etwa einem Fünftel der Fälle (*n* = 23) oberhalb 50 %, Abb. 1c.

### Postoperative Audiometrie

**Figure 2:**
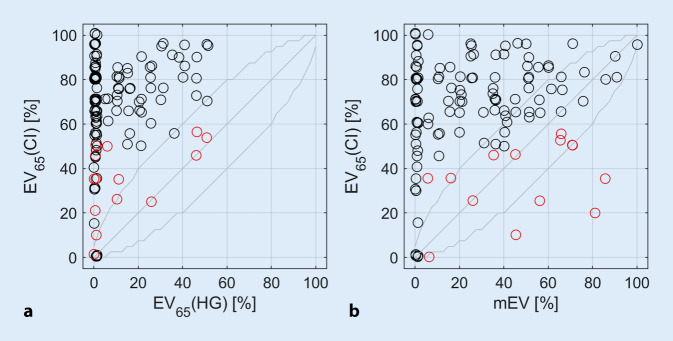

Die Abb. 2 zeigt die Sprachverstehenswerte mit CI, EV_65_(CI), gemessen nach 6 Monaten Hörerfahrung mit CI, in Abhängigkeit vom präoperativen EV_65_(HG) (Abb. 2a) bzw. mEV (Abb. 2b). Das mittlere Sprachverstehen verbesserte sich nach 6 Monaten von 10 % mit HG auf 65 % mit CI. In 90 % der Fälle (*n* = 112) verbesserte sich das Einsilberverstehen um mindestens 20 Prozentpunkte (%-Punkte). Eine im Einzelfall statistisch signifikante Verbesserung des Einsilberverstehens wurde für 93 % der Fälle (*n* = 115) nach 6 Monaten beobachtet. Die Prüfung der Signifikanz erfolgte anhand der kritischen Differenzen nach Winkler und Holube [10]. Für keinen der Fälle wurde eine Verschlechterung des Einsilberverstehens beobachtet. In 116 Fällen lag das Einsilberverstehen mit CI, EV_65_(CI), innerhalb des Vertrauensbereichs des Freiburger Tests oder besser als das präoperative mEV. In 8 Fällen war das EV_65_(CI) signifikant [10] geringer als das mEV.

**Figure 3:**
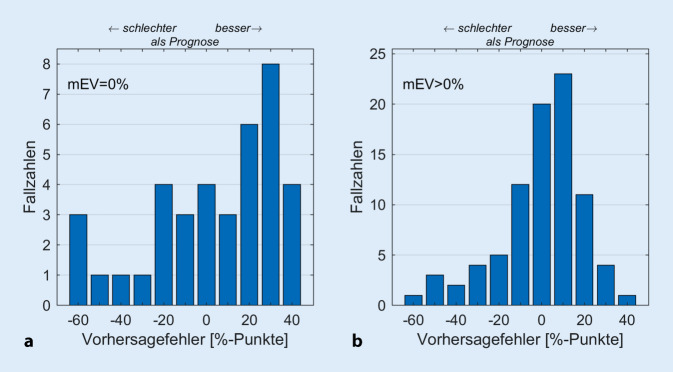

Die Abb. 3 zeigt die Differenzen zwischen dem nach 6 Monaten postoperativ gemessenen EV_65_(CI) und dem für diesen Zeitpunkt nach Gl. 1 vorhergesagten Wert für 2 Subpopulationen, mEV = 0 % und mEV > 0 %, dieser Studie. Diese Einteilung wird durch eine frühere Studie motiviert [13]. In Abb. 3a sind die Differenzen der 39 Patienten mit einem präoperativen maximalen Einsilberverstehen von Null (mEV = 0 %) zusammengefasst. Positive Werte entsprechen einem besser als vorhergesagten Verstehen. Der mediane absolute Fehler für die Vorhersage liegt hier bei 23,2 %-Punkten. Es findet sich keine Korrelation zwischen vorhergesagtem und gemessenem Sprachverstehen (*p* > 0,23). Die Abb. 3b zeigt die Zusammenfassung der Differenzen der 85 Patienten mit einem präoperativen mEV, das besser als Null war (mEV > 0 %). Der mediane absolute Fehler für die Vorhersage liegt hier bei 11,5 %-Punkten. Für 47 Fälle lag der Fehler in einem Korridor von ± 10 %-Punkten, 32 Fälle unterschritten die Vorhersage um mehr als 10 %-Punkte, während für 45 Fälle ein um mehr als 10 %-Punkte über der Vorhersage liegendes Sprachverstehen beobachtet wurde.

**Figure 4:**
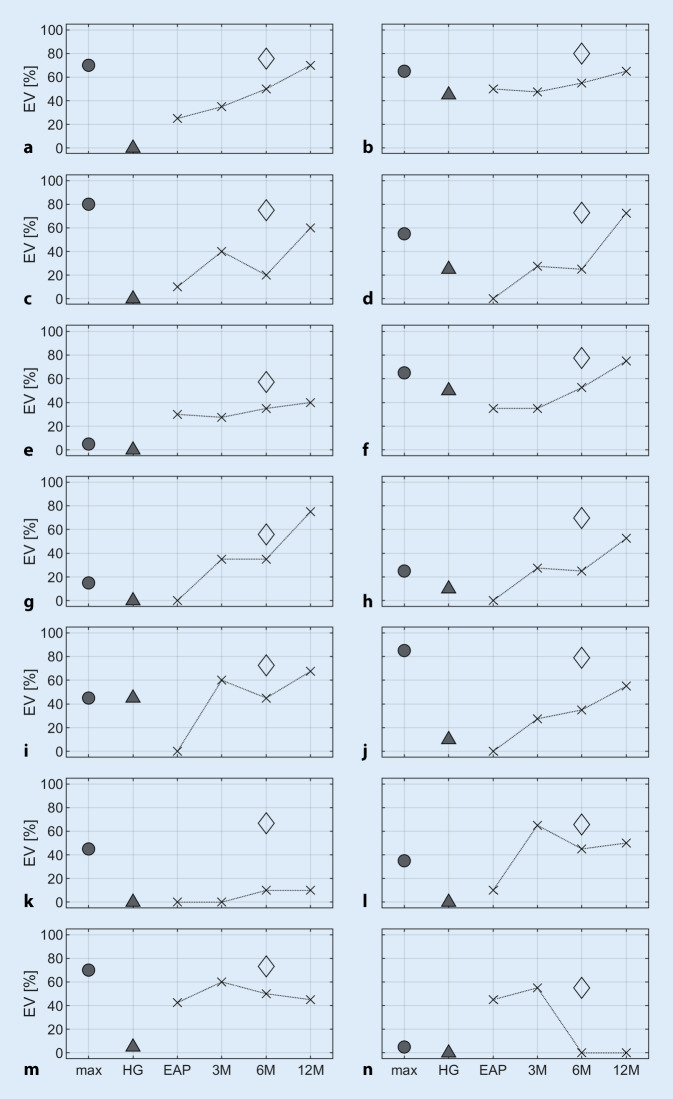

In Abb. 4 sind ausgewählte Einzelfälle aus der in Abb. 3b zusammengefassten Subpopulation in ihrem zeitlichen Verlauf dokumentiert. Dargestellt sind hier alle Fälle aus Abb. 3b, welche das vorhergesagte Sprachverstehen um mehr als 20 %-Punkte verfehlt haben. Diese werden im Folgenden als Fälle mit *unerwartet schlechtem Sprachverstehen* bezeichnet. Von diesen insgesamt 14 Fällen erreichten 9 (Abb. 4a–i) nach 12 Monaten das prognostizierte Sprachverstehen innerhalb eines Fensters von 20 %-Punkten. Ein weiterer Fall (Abb. 4j) zeigt einen verlangsamten Anstieg des Sprachverstehens, welcher bei fortschreitender Folgetherapie ein verspätetes Erreichen der Prognose erwarten lässt. In den verbleibenden 4 von 85 Versorgungen (4,7 %) mit einem präoperativen mEV > 0 % ist aufgrund eines sehr flachen Anstiegs (Abb. 4k) oder aber aufgrund eines mäßig (Abb. 4l, m) bis stark (Abb. 4n) fluktuierenden Sprachverstehens keine Verbesserung des Sprachverstehens absehbar.

## Diskussion

In dieser prospektiven Studie verbesserte sich das mittlere Sprachverstehen nach 6 Monaten von 10 % mit HG auf 65 % mit CI. Hier wurde eine signifikante [10] Verbesserung für 93 % der Fälle beobachtet. In keinem der Fälle trat eine Verschlechterung des Sprachverstehens nach CI-Versorgung auf. Darüber hinaus wurde ein in einer vorhergehenden retrospektiven Studie [14], Gl. 1, entwickeltes Modell zur Vorhersage des Einsilberverstehens nach 6‑monatiger CI-Rehabilitation evaluiert. Der Vorhersagefehler lag in den Fällen mit präoperativem residualem Einsilberverstehen (mEV > 0 %) bei 11,5 %-Punkten und bei solchen mit mEV = 0 % bei 23,2 %-Punkten.

### Wesentliche Parameter

Initial war das Modell anhand der Daten von Patienten mit präoperativem Hörverlust, der besser als 80 dB war, entwickelt worden. Für die Mehrheit (92 %) wurde damals ein mEV > 0 % gemessen. Daher erscheint es zunächst sinnvoll, sich bei einer Erweiterung des Anwendungsbereichs in Richtung höherer Reintonhörverluste auf Fälle mit mEV > 0 % zu begrenzen. Für diese Gruppe (*n* = 85) ist eine zumindest minimale Funktionalität des Hörnervs gegeben. Hier wurde lediglich in 4 Fällen auch längerfristig ein unerwartet schlechtes Sprachverstehen beobachtet, das vermuten lässt, dass das präoperativ prognostizierte Sprachverstehen nicht erreicht wird. Hingegen liegt in der Gruppe mit mEV = 0 % (*n* = 39) keine derartige Information über die Hörnervfunktion vor. Daher ist der Prädiktionsfehler für diese Gruppe erwartbar größer.

Das mEV wurde in einer früheren Arbeit als Minimalprädiktor für das EV_65_(CI) eingeführt [13]. In der aktuellen Patientenpopulation wird das mEV in 116 Fällen (93,5 %) erreicht oder übertroffen. In 8 Fällen (6,5 %) ist das EV_65_(CI) signifikant [10] geringer als das mEV. Insgesamt zeigt sich hier eine weitgehende Übereinstimmung mit einer andernorts durchgeführten Studie [27], welche auch auf die große Bedeutung des mEV im Rahmen der CI-Versorgung hinweist.

In einer Studie von Shafieibavani et al. [26] wurden verschiedene Modellansätze miteinander verglichen. Die Autoren geben mittlere Vorhersagefehler von 20–22 %-Punkten an. Diese Analyse basierte auf 2489 Fällen, welche zwischen 2003 und 2018 entweder in einer deutschen, einer US-amerikanischen oder einer australischen Einrichtung versorgt worden sind. Die publizierten präoperativen audiometrischen Befunde legen nahe, dass es sich hierbei zu einem großen Teil um Patienten handelte, die ein mEV nahe Null aufgewiesen hatten. Die dort gefundenen Modellfehler liegen hierzu passend in der Größenordnung der in Abb. 3a dargestellten Vorhersagefehler mit einem MAE von 23,2 %-Punkten für die Patientengruppe mit mEV = 0 %.

Der gefundene MAE von 11,5 %-Punkten für die Population mit einem mEV > 0 % rechtfertigt post hoc die hier beschriebene Anwendung des Vorhersagemodells [14]: Die Begrenzung der Population auf funktionelles Restgehör im Sinne eines noch messbaren Sprachverstehens trägt erheblich zu der Verringerung des Vorhersagefehlers bei. In der vorherigen Studie [14] bestand diese Begrenzung im Einschlusskriterium auf Fälle mit einem Hörverlust von höchstens 80 dB_HL_. In dieser Gruppe ist das mEV in aller Regel größer als Null [15]. Bei Kandidaten mit einem Hörverlust von mehr als 80 dB_HL_ wurde für 44 von 82 Fällen (54 %) ein mEV > 0 % beobachtet. Diese Fälle sind in Abb. 4b enthalten. Zusammenfassend ist also die Modifikation des ursprünglichen Anwendungsbereichs des Vorhersagemodells für Hörverluste von höchstens 80 dB_HL_ auf das Sprachverstehen von mEV > 0 % gerechtfertigt [14], da der MAE nahezu unverändert bleibt. Die Anwendung auf Fälle mit mEV = 0 ist möglich, jedoch mit einem größeren Modellfehler verbunden (Abb. 3a). Über Gl. 1 wird das prognostizierte Einsilberverstehen für diese Fälle durch die Konstante β_0_ und das Lebensalter determiniert. Die Konstante β_0_ repräsentiert das mittlere Sprachverstehen mit CI ohne die individuellen Korrektureinflüsse der anderen 3 Variablen.

### Qualitätssicherung in der CI-Therapie

Der hier vorgestellte Vorhersagewert kann gemeinsam mit dem mEV als Qualitätssicherungsparameter genutzt werden. Dadurch ergibt sich ein Korridor, innerhalb dessen das postoperative Einsilberverstehen mit CI liegen sollte. Die Abweichung vom Vorhersagewert in Kombination mit der Abweichung vom unteren Erwartungswert, dem mEV [13], ermöglicht eine frühzeitige Identifikation von Fällen mit *unerwartet schlechtem Sprachverstehen*, Abb. 4, und die Einleitung entsprechender zusätzlicher Maßnahmen im Rahmen der Basis- und Folgetherapie. Zunächst sind pathophysiologische Ursachen und technische Fehlfunktionen [2] auszuschließen. Im weiteren kommen zusätzliche technische Prozessoranpassungen bzw. die Modifikation und Intensivierung der Hör- und Sprachtherapien, aber auch die Überprüfung des Nutzerverhaltens und entsprechende Beratung [6, 7, 21] in Betracht. Im Rahmen der Nachbehandlung der hier vorgestellten Fälle wurde eine solche Qualitätssicherung durchgeführt und nach 3 Monaten CI-Erfahrung das EV_65_(CI) mit dem präoperativen mEV und dem Vorhersagewert nach Gl. 1 verglichen. In einer interdisziplinären Fallbesprechung wurden dann ggf. ergänzende Therapiemodifikationen eingeleitet. Dies könnte dazu geführt haben, dass die tatsächlich erreichten Sprachverstehensleistungen leicht oberhalb der Vorhersage lagen (Abb. 3).

Das hier vorgestellte Modell ist für Fälle mit mEV > 0 mit einem geringen Vorhersagefehler verbunden. Dies trifft in der untersuchten Population auf etwa 2/3 aller postlingual ertaubten erwachsenen CI-Kandidaten zu. Gerade diese Gruppe hat nachvollziehbare Bedenken, sich einer für die CI-Versorgung notwendigen Operation zu unterziehen. Insofern ist für diese CI-Kandidaten die Abschätzung des Versorgungserfolgs besonders nützlich.

Das Vorhersagemodell wurde zwar anhand der Daten von Patienten mit CI-Systemen eines Herstellers entwickelt, die gefundenen Abhängigkeiten sollten sich aber auch auf Versorgungen mit anderen Systemen anwenden lassen. Zur Bestimmung der quantitativen Abhängigkeiten für unterschiedliche CI-Systeme und Rehabilitationskonzepte wären weitere Studien an anderen Einrichtungen wünschenswert. Prinzipiell ist es anzustreben, dass die Prognose des Sprachverstehens mit CI auf der Basis präoperativer Daten beruht. Neben den hier vorgestellten Werten könnten auch noch Ergebnisse zukünftiger hördiagnostischer Verfahren [20] zur Modellbildung beitragen. Darüber hinaus könnten auch intraoperative Messungen genutzt werden und somit der Qualitätssicherung dienen. Hierzu sind weitere Studien notwendig.

## Fazit für die Praxis

- Die Cochleaimplantat(CI)-Versorgung auch von Patienten mit einem mittleren Hörverlust in der Größenordnung von 60 dB_HL_ und unzureichendem Sprachverstehen mit Hörgerät stellt eine Therapieoption dar.
- Das auf präoperativ erhobenen Daten basierende Modell zur Vorhersage des Sprachverstehens mit CI kann bei der Beratung von CI-Kandidaten und für die Qualitätssicherung in der postoperativen Rehabilitation verwendet werden.
- Die Begrenzung auf eine Population mit präoperativem Einsilberverstehen, das besser als Null ist, verringert den Vorhersagefehler.
- Das Modell erlaubt die frühzeitige Identifizierung von Fällen mit unerwartet schlechtem Sprachverstehen.
